# Perceptions of Schoolteachers About Teledentistry Use in Makkah City

**DOI:** 10.7759/cureus.51927

**Published:** 2024-01-09

**Authors:** Alaa Husni Qari, Shiamaa S Almashhadani, Muhnnad A Balbaid, Yasir D Alharthi, Ahmed A Alzahrani, Majd H Morad, Sherif S Hassan

**Affiliations:** 1 Preventive Dentistry, Umm Al-Qura University, Makkah, SAU; 2 Public Health Dentistry, Dubai Health, Dubai, ARE; 3 Dentistry, Umm Al-Qura University, Makkah, SAU; 4 Basic and Clinical Oral Sciences, Umm Al-Qura University, Makkah, SAU

**Keywords:** school age children, schoolteachers, perception, telemedicine, teledentistry

## Abstract

Objectives: Teledentistry is a combination of communication technology and dentistry. Teledentistry is an alternative and useful way to remotely provide advisory, preventive, and emergency services in places with poor access to dental care. Few studies in Saudi have investigated the implementation of teledentistry at schools or assessed the perceptions of school personnel. This research aimed to evaluate teachers' perceptions about the use of teledentistry to examine schoolchildren in Makkah city.

Methods: The study was conducted on 20 intermediate schools randomly selected from different regions of Makkah city using a multistage cluster sampling method. Two data collectors visited the schools to administer the study questionnaire, exploring teachers' perceptions of teledentistry. The questionnaire was adapted from a validated survey based on the technology acceptance model (TAM). Descriptive statistics, analysis of variance, Mann-Whitney U test, and the Kruskal-Wallis tests were conducted to compare the mean differences in participants' perceptions of teledentistry based on their demographics.

Results: A total of 241 teachers completed the survey, 131 of whom were females (54.4%), and the mean age of participants was 42.5±7 years. Over 80% of participants taught ≥16 classes a week, and their experience ranged from less than a year to more than 31 years. Sixty participants (25%) knew teledentistry before watching the study video. The results revealed a significant difference between male and female teachers regarding perceptions of teledentistry. Female participants had higher scores in TAM dimensions than males.

Conclusion: This survey revealed a generally favorable attitude toward teledentistry utilization in schools. Including non-dental employees, such as teachers to provide such a service will assist in alleviating the shortage or unavailability of dentists at schools. This goes in line with the government's plans to promote telehealth as part of the Saudi Vision 2030.

## Introduction

Telemedicine refers to the use of electronic transmission of medical data from a remote location to a physician to improve a patient's clinical health condition [1]. Teledentistry is a branch of telemedicine that has a significant impact on oral health services. It utilizes digital and communication technology to provide dental care, consultation, and education remotely without the need for face-to-face interaction with service recipients [2]. Teleconsultation, telediagnosis, tele-triage, and telemonitoring are essential services in dental practice, particularly in rural areas [2]. Using these applications, access to dental services, including advisory, preventive, and emergency care, can be provided to underprivileged populations [1]. Teledentistry has the potential to identify at-risk groups, facilitate referrals to dental specialists, and improve local treatment. This can help reduce waiting lists, travel time, and loss of productivity [2].

The dentist is the first health professional to discover oral health issues in schoolchildren, including tooth decay, misaligned teeth, and parafunctional habits [3]. Subsequently, an appropriate treatment plan is developed for children during their first dental visit [3]. However, 38.3% of schoolchildren in Saudi Arabia have never visited a dentist, while 30.3% only seek dental care when they are experiencing dental pain [4]. The unequal distribution of pediatric dentists in Saudi Arabia or their unavailability in some cities has added difficulty for children to access dental care [5]. Teledentistry can address this barrier by providing children with remote access to dental care services [6]. Several studies have evaluated teledentistry applications in various settings, including schools, and have supported its use, especially for the early detection of dental caries and oral diseases [7,8]. Teledentistry can be used as an alternative screening method for dental caries, as well as for remote consultation and treatment planning [7]. It can also help detect child abuse, as it is a common problem among children [5].

Smartphone devices can be useful for detecting caries in schools, as they are widely available and affordable [9]. The engagement of non-dental personnel, such as school nurses or teachers, with teledentistry, is believed to be a reliable and cost-effective strategy that can help overcome the shortage or unavailability of dentists [10]. Implementing teledentistry could increase the rate of dental care services provided at schools through remote monitoring and evaluation of oral health among children, without the physical presence of a dentist [11,12].

Limited research exists about teledentistry in Saudi Arabia [9,13]. Studies assessed the knowledge and awareness of dental providers and students towards teledentistry [13-15]. The results showed a positive attitude towards the implementation of it in dental practice, specifically regarding diagnosis and education [13-15]. Another study highlighted the accuracy of remotely detecting dental caries among schoolchildren, where non-dental staff captured intraoral images using smartphone devices [9]. This screening method has been proven to be accurate and cost-effective [9]. In the study conducted by Whitten et al. in 2000, researchers examined the possible involvement of school staff in utilizing telemedicine and assessed their attitudes toward its implementation in schools [16]. The results showed a negative attitude among school staff towards implementing telemedicine at schools [16]. This challenge, however, was overcome when they witnessed firsthand the use of this technology in serving school children [16]. Our study aimed to evaluate teachers’ perceptions of the use of teledentistry in schools in Makkah city and how these perceptions are associated with their demographic characteristics.

## Materials and methods

Ethical consideration

Ethical approval was obtained from the Biomedical Research Ethics Committee at Umm Al-Qura University (UQU) (Approval No: HAPO-02-K-012-2022-11-1313).

Study design and sample population

This cross-sectional study was conducted on schoolteachers of the intermediate schools in Makkah city between November 2022 and February 2023. Schools were selected from different parts of Makkah using a multistage cluster sampling technique. About 20 intermediate schools were selected, 10 of which were for boys and the remaining were for girls. A convenient sample of teachers from the selected intermediate schools was included in this study. Two data collectors, one male and one female, approached the school’s administration and then individually reached out to schoolteachers during break time or a time designated by the administration. A self-reported questionnaire was administered to 525 participants, and a total of 241 teachers completed the survey (response rate: 45.90%). Participants who didn’t sign the study consent form were excluded.

Questionnaire development

The study questionnaire was adapted from a validated survey based on the technology acceptance model (TAM) [17]. This theory intends to understand the behavioral aspect of technology use (i.e., factors affecting people's acceptance of a technology) [18]. The questionnaire was translated into the Arabic language and reviewed by dental experts, two public health specialists, and one dental science specialist. A pilot test was conducted to assess the understanding of the questionnaire. A total of 12 participants completed the pilot testing and modifications were made to the survey. This included a further clarification of the survey items and the accompanying video, rearrangement of the survey statements, addition of other school subjects for teachers, cutting off the length of the video, and adding an open question at the end of the survey inquiring about participants' opinions on teledentistry application at schools.

The questionnaire included four sections: 1) the consent form; 2) participants’ demographic data, including gender, age, years of experience, job rank, specialty, school name, number of weekly classes, and smartphone use; 3) an awareness video about teledentistry; 4) participants perceptions about teledentistry. The awareness video explained the prevalence of tooth decay among schoolchildren in Makkah, and the obstacles encountered by the UQU mobile clinic to reach schools in rural areas. The video also talked about the potential role of non-dental specialists such as schoolteachers in implementing teledentistry at schools and explained the various applications and advantages of teledentistry for children. It showed a live example of a school staff taking intraoral photos of kids. The fourth section of the survey contained 16 questions, covering the following categories of TAM: 1) Perceived ease of use (PE): a person's belief that using a specific system will be effortless [17]. 2) Perceived usefulness (PU): a person's belief that using a specific system will improve their ability to perform their job [17]. 3) Attitude toward a behavior (ATB): a person’s feelings, whether positive or negative, towards performing a targeted behavior (i.e., using a specific system) [17]. 4) Anxiety (ANX): inducing anxiety or emotions when performing a targeted behavior [17]. 5) Self-efficacy (SE): a belief in one’s ability to utilize a specific system to accomplish a job or task [17]. Participants’ responses were based on a five-point Likert Scale; each question had five possible responses that ranged from 1 to 5, with 1 indicating "strongly disagree" and 5 indicating "strongly agree." The higher value indicates a positive attitude towards teledentistry.

Statistical analysis

Descriptive statistics tests were conducted to assess participants’ perspectives toward teledentistry use in schools. The relationship of TAM dimensions with participants’ demographic characteristics was evaluated using Kruskal Wallis and Mann-Whitney U tests. Cronbach’s alpha (α) was calculated to test the reliability of the questionnaire items. A p-value of 0.05 was used for statistical differences. The IBM SPSS Statistics for Windows, Version 29 (Released 2022; IBM Corp., Armonk, New York, United States) was used for analyses.

## Results

Characteristics of participants

The survey was distributed to 20 schools, two schools were excluded because of no response from the school administration or lack of cooperation. The questionnaire was administered to 525 participants from 18 schools, and a total of 241 teachers completed the survey (response rate 46%). Most responses came from the Middle region of Makkah (n=61, 25%) followed by the East region (n=58, 24%), and 131 of the sample were females (54.4%) (Table 1). The mean age of participants was 42.5±7 years. Over 80% of participants taught ≥16 classes a week (n=195), and the subjects that were taught the most were art and social studies (n=100, 41.5%) (Table 1). Teachers’ work experience ranged from less than a year to more than 31 years (Table 1). About 60 participants (25%) knew what teledentistry was before watching the video, and all participants used smartphones.

**Table 1:** Characteristics of schoolteachers in intermediate schools in Makkah city (n=241)

Characteristics		No.	(%)
Gender	Male	110	45.6
	Female	131	54.4
Years of experience	0-10 years	42	17.4
	11-20 years	116	48.1
	21 and more	83	34.4
Subject	Science	86	35.7
	Islamic	43	17.8
	Art and social studies	100	41.5
	Other	12	5
Job rank	Teacher	53	22
	Practice teacher	168	69.7
	Advanced/expert teacher	20	8.3
Number of classes	<16 classes/week	46	19.1
	≥16 classes/week	195	80.9
Region	Middle	61	25.3
	East	58	24.1
	West	38	15.8
	North	42	17.4
	South	42	17.4

Descriptive statistics

The teachers' perspectives towards the use of teledentistry in schools were demonstrated by the mean and standard deviation. The values for each dimension of TAM ranged from 1 (strongly disagree) to 5 (strongly agree). The means of all dimensions were greater than 3, indicating that schoolteachers have a positive attitude toward the use of teledentistry in all TAM dimensions. The results are the following: ATB (4.47 SD+0.7) received the highest scores, followed by ANX (4.19 SD+0.6), PU (4.32 SD+0.8), PE (4.31 SD+0.7), and SE (3.98 SD+0.9).

Reliability assessment

This study used Cronbach’s alpha (α) to assess the consistency of TAM dimensions items and how they correlate with each other. The reliability test was excellent for all the TAM dimensions (Cronbach alpha>0.80) except for ANX, which had the lowest reliability score (Cronbach alpha=0.67). However, this item still had an acceptable reliability (Table 2).

**Table 2:** TAM dimensions and its measuring scale items TAM: Technology acceptance model; PE: Perceived ease of use; PU: Perceived usefulness; ATB: Attitude toward a behavior; ANX: Anxiety; SE: Self-efficacy

Constructs	Items	Cronbach’s (α)
PE1	1) My interaction with teledentistry would be clear and understandable.	0.809
PE2	2) Learning to use teledentistry for dental examinations would be easy for me.	
PE3	3) It would be easy for me to become skillful at using teledentistry.	
PU1	1) Using teledentistry could improve the dental care provided to students.	0.854
PU2	2) If I were to use teledentistry, I could use it in a dental emergency.	
PU3	3) Using teledentistry would enable me to accommodate students’ dental needs more quickly.	
ATB1	1) Using teledentistry for students’ dental assessment is a good idea.	0.803
ATB2	2) Teledentistry would make student dental screening more interesting.	
ANX1	1) I think dental professionals can adequately make a dental assessment of students when not being physically present.	0.668
ANX2	2) More research is needed on the effectiveness of teledentistry before I would use it for students.	
ANX3	3) I do not like the loss of personal contact with dentists associated with using teledentistry.	
ANX4	4) I hesitate to use teledentistry for fear of making mistakes I can’t correct.	
SE1	1) I could use teledentistry even if I had never used this technology before.	0.803
SE2	2) I could use teledentistry if someone showed me how to use it beforehand.	
SE3	3) I could use teledentistry even if there was no one around to tell me what to do as I go.	

Relationship of TAM with demographics

There was no association between the perspectives of school personnel regarding the use of teledentistry based on their demagogic except for the gender. The results revealed a significant difference in perceptions towards teledentistry use between male and female teachers. Female participants had higher scores in TAM dimensions than males (Table 3).

**Table 3:** Perspectives among male and female schoolteachers PE: Perceived ease of use; PU: Perceived usefulness; ATB: Attitude toward a behavior; ANX: Anxiety; SE: Self-efficacy

	Male	Female	p-value
	Mean rank		
PE	106.7	133.1	0.003
PU	105.1	134.4	<0.001
ATB	108.8	131.2	0.007
ANX	103.1	136.1	<0.001
SE	104.9	134.5	<0.001

## Discussion

Teledentistry is an alternative and valuable method for delivering advisory, preventive, and emergency dental services to areas where finding a dentist is challenging [19]. It involves exchanging clinical information and images of an individual over long distances for a dental consultation or follow-up care [19]. Using teledentistry in school settings can help provide access to dental care, especially to children from various socioeconomic levels [20]. School-based programs are more effective in delivering dental care as they save time and money as well as prevent children from unnecessary absences from school [20,21]. Also, in such programs, teachers could customize oral health messages and instructions for children due to their close connection and daily influence on them in the classroom [21]. As shown in past studies, the application of teledentistry in schools by non-dental personnel can positively impact public dental health [9]. A pediatric teledentistry program in the US has presented the effect of including community health workers and care coordinators with the teleconsultation team on kids' oral health [22,23]. Their role has increased children's compliance with dental treatment and improved their access to dental care [22,23].

The personal connection between schoolteachers and children would signify their potential role to be part of the dental team, using teledentistry, to deliver oral health care at schools. This as a result would improve children’s oral health and well-being [21]. However, to our knowledge, limited research exists about teachers’ perceptions of teledentistry use in schools.

Our study indicated that teachers have a positive attitude towards using teledentistry in schools. It also highlighted a significant difference between male and female teachers regarding their perceptions of teledentistry. Female participants scored higher on the TAM dimensions than male participants. This is in agreement with past studies, that gender significantly influences the perceptions of using a new technology [18]. Similar to Goswami and Dutta in 2015, female participants were more anxious than males in using technology [18]. However, in his study, women had lower self-efficacy in using technology than men [18]. In other studies, females had also lower PE compared to men due to their nervousness with technology [18]. Both differ from the results of this study whereas SE and PE are higher among female teachers than males. This might be due to this study's accompanying video. The video showed how teledentistry, with training, can be used by non-dental personnel in school settings. Ammenwerth et al. stated that training might positively influence participants’ PE and PU and therefore increase their acceptance of using technology - the nursing electronic health records [24]. This study video might have also influenced the high scores among female teachers over males because of the social influence component of its content. A review by Goswami and Dutta in 2015 stated that social influence had a greater impact on female acceptance of new technologies than their personal choice, while social influence had less effect on male acceptance of technologies [18]. Training and videos could influence people's acceptance of technologies. However, further studies are needed to confirm this relationship regarding using teledentistry.

Engaging non-dental personnel to provide teledentistry services can be reliable and reduce cost and effort. A study found that using readily available smart devices by non-dental staff can generate diagnostic images that effectively motivate children to prioritize their dental health [25]. Researchers found that both dental and non-dental teledentistry examinations demonstrated strong and nearly perfect reliability in detecting dental caries in primary teeth [9,26]. However, when it came to detecting caries in permanent teeth, the reliability was strong in the non-dental teledentistry examination and moderate in the dental teledentistry examination [27]. Photos captured by non-dental personnel showed an excellent level of comfort among children [27]. This was reflected positively in their cooperation [27].

The strengths of this study are displayed in various aspects. Researchers utilized a validated survey (TAM) and conducted a pilot test with participants to ensure accurate results. In addition, the study sample was selected from various regions of Makkah city using a multi-cluster method to ensure representative results. However, compliance and collaboration among participants were identified as key issues and presented as challenges to this research. Some teachers did not watch the entire video demonstrating the implementation of teledentistry. Also, a typical limitation in this type of study is the response bias. The survey relies on participants’ self-reported data, which might have introduced some inaccuracies in our results. To overcome these limitations, data collectors were assigned to individually contact and assist schoolteachers with the surveys. Despite the study challenges, participants’ responses were generally positive towards teledentistry.

As mentioned earlier, females exhibited a more favorable attitude towards the adoption of teledentistry in schools across all dimensions of the TAM model. Therefore, it is recommended to establish teledentistry for remote oral health services at schools that have a predominantly female teaching staff. Another recommendation is to emphasize the importance of educating and training the teaching staff to ensure a positive attitude and collaboration when implementing such services for schools.

## Conclusions

In conclusion, the findings of this survey underscore a positive sentiment toward the adoption of teledentistry within school environments. The application of the TAM in our study has highlighted a strategic avenue for addressing the scarcity of dental professionals within schools. Specifically, the incorporation of non-dental staff, such as teachers, in the delivery of dental services emerges as a promising solution to bridge the gap in dental care accessibility. This approach not only mitigates the challenge of dentist shortages but also contributes to the enhancement of oral health literacy within the community. By targeting school staff, it becomes possible to provide oral health messaging among students, aligning with the broader governmental agenda of promoting telehealth as an integral component of Vision 2030. Therefore, this study advocates for the integration of teledentistry, leveraging the collaboration of diverse stakeholders, to increase both the reach and impact of oral health initiatives in school settings.
